# A Survey of Pharmacogenomics Testing Among Physicians, Pharmacists, and Researchers From China

**DOI:** 10.3389/fphar.2021.682020

**Published:** 2021-07-12

**Authors:** Chengxian Guo, Biwen Hu, Chengjun Guo, Xiangguang Meng, Yun Kuang, Longjian Huang, Danling Wang, Kangwei Xu, Yanlin Zhao, Guoping Yang, Weimin Cai, Yan Shu

**Affiliations:** ^1^ Center of Clinical Pharmacology, The Third Xiangya Hospital, Central South University, Changsha, China; ^2^ School of Applied Mathematics, Guangdong University of Technology, Guangzhou, China; ^3^ Laboratory of Cardiovascular Disease and Drug Research, Zhengzhou No. 7 People’s Hospital, Zhengzhou, China; ^4^ Youjiang Medical University for Nationalities, Baise, China; ^5^ Department of Neuroscience, Hengyang School of Medicine, University of South China, Hengyang, China; ^6^ Changsha Central Hospital, University of South China, Hengyang, China; ^7^ Hunan Normal University School of Medicine, Changsha, China; ^8^ Department of Clinical Pharmacy, School of Pharmacy, Fudan University, Shanghai, China; ^9^ Department of Pharmaceutical Sciences, School of Pharmacy, University of Maryland at Baltimore, Baltimore, MD, United States

**Keywords:** pharmacogenomics, pharmacogenetics, genomics, survey, clinical Pharmacology

## Abstract

To elucidate current domestic factors influencing pharmacogenomics (PGx) implementation and its future in China, we conducted a questionnaire survey on PGx applications and testing. A questionnaire-based survey was created on the popular online professional survey platform “Wenjuanxing” (www.wjx.cn) and performed *via* the social media platform WeChat. Among 422 participants, there were physicians (27.7%), pharmacists (31.3%), and researchers (41.0%). We found that less than 50% of physicians were aware of the importance of PGx in drug therapy, while over 50% of pharmacists and researchers recognized the importance. Only 38.5% of physicians, 40.9% of pharmacists, and 55.5% of researchers concurred that PGx analysis could lower the economic burdens for patients. However, most of the responders affirmed that PGx should be effectively implemented in clinical practices. A lack of sector standards, a lack of clinical research, and a lack of guidelines were found to be the major factors for hindering PGx clinical application. Among drugs associated with PGx assays, the most common were warfarin and clopidogrel. Although PGx research has advanced rapidly in recent years in mainland China, the clinical implementation of PGx has a long way to go.

## Introduction

It has been widely confirmed that PGx may impact drug therapeutic efficacy and adverse responses ^[1∼3]^. The advances of PGx have accelerated practice in precision medicine and individualized therapy (Roden et al., 2019). At present, the United States Food and Drug Administration (FDA) recommends PGx assays to be performed while prescribing approximate 260 drugs (Table of Pharmacogenomic Biomarkers in Drug Labeling, 2021). The authoritative PGx database PharmGKB has complied gene annotations for 685 drugs, 149 drug-related pathways, and 143 clinical guidelines (PharmGKB, 2021). A variety of global organizations, such as the United States Clinical Pharmacogenetics Implementation Consortium (CPIC^®^), the Dutch Pharmacogenetics Working Group (DPWG), the Canadian Pharmacogenomics Network for Drug Safety (CPNDS), and the French National Network of Pharmacogenetics (RNPGx) have established relevant clinical guidelines for PGx assays (Clinical Pharmacogenetics Implementation Consortium (CPIC), 2021; Swen et al., 2008; Canadian Pharmacogenomics Network for Drug Safety, 2021; Picard et al., 2017). Many US hospitals, including Mayo Clinic, Mount Sinai Medical Center, St. Jude Children’s Hospital, College of Medicine of Florida University, and Medical Center of Vanderbilt University, have implemented PGx-guided dosing protocols in clinical practice (Dunnenberger et al., 2015).

In the mainland of China, the development of PGx has also achieved a rapid progress. In 2011, the Chinese Pharmacological Society established the Division of Pharmacogenomics focus on the research and applications of PGx in the mainland of China (Chinese Pharmacological Society, 2011). In 2015, the Chinese Ministry of Health published the *Interim Guidelines of Detection Techniques for Drug Metabolizing Enzymes and Acting Target Genes* and the *Interim Guidelines of Detection Techniques of Individualized Antineoplastic Therapies* (National Health Commission Of the People's Republic of China, 2015). Both guidelines highlighted the necessity of standardizing and codifying PGx detections. At present, National Medical Products Administration have endorsed approximate 200 dosing-related reagent kits for PGx testing (National Medical Products Administration, 2021). However, the translation of PGx into clinical practices has been relatively slow. Although an increasing number of Chinese companies and hospitals have implemented PGx testing, the volume of annual assays is still limited (Guo et al., 2018). The popularization of PGx is closely associated with the scientific progresses by advocating researchers and the interpretations of patient implementation by physicians. For elucidating the hindering factors of PGx's clinical application, we have referenced multiple PGx-related questionnaires with some modifications (Stanek et al., 2012; Bank et al., 2017; Just et al., 2017). In the present study, a questionnaire of PGx has been designed to obtain a comprehensive understanding of PGx by healthcare providers and researchers. We aimed to provide practical guiding information for future development of PGx guidance in the mainland of China.

## Methods

The questionnaire was designed consisting of 20 questions in five major categories (Supplementary materials). Questions 1–5 (Supplementary Questions Q1–Q5) were the basic profiles of the participants, including gender, age, educational level, occupation, and residence. The surveyed knowledge of PGx (Supplementary Questions Q6–Q10) included whether or not PGx guides in selecting optimal drugs, choosing correct drug doses, and predicting adverse drug reactions. A multi-choice question then asked the opinions from the participants on the main factors hindering the clinical application of PGx in China (Supplementary Question Q11). (Supplementary Questions Q12–Q19) was to inquire the measures and strategies to promote clinical application of PGx. Lastly, we asked specific PGx testing at the responders institutions (Supplementary Question Q20). For most survey questions (Supplementary Questions Q6–Q10) and (Supplementary Questions Q12–Q19), five choices were given as *do not know at all; disagree; hard-to-say and inclined-to-disagree*; *hard-to-say and inclined-to-agree*; *agree*. As compared to other questionnaires, two options of *hard-to-say and inclined-to-disagree* and *hard-to-say and inclined-to-agree* were added. This was to clearly differentiate agree and disagree responders and to avoid assigning those only with an inclination into agree or disagree responders. The questionnaire was uploaded to the popular online professional survey platform what is named “Wenjuanxing” (www.wjx.cn) for data collection questionnaire surveys. The survey link online was conducted *via* the social media platform WeChat. The surveying period was from April to May 2019. The major responders included physicians, pharmacists, and researchers. Participants were selected as physicians, pharmacists, and researchers. The exclusion criterion was participants who are not one of the above-mentioned professionals. The study protocol was approved by the Institutional Review Board (AAHRPP-accredited) of the Third Xiangya Hospital of Central South University. Participation was voluntary, and all responders remained anonymous.

### Data Processing

The quantitative data were analyzed by percentages. Descriptive analysis was performed for all of the participants, who were stratified into three groups for comparison: physicians, pharmacists, and researchers. When applicable, the attitude toward a question with an agreement rate over 50% was deemed as positive and below 50% as negative, respectively. If there was positive and negative among the three groups, the differences were further evaluated by statistical analysis. A chi-square test was conducted to determine whether statistical differences existed question the answers among physicians, pharmacists, and researchers. The confounding factors were analyzed by logistic regression analysis. The statistical significance of logistic regression method was evaluated using adjusted OR, 95%CI, and *p* values. *p* < 0.05 was considered statistically significant. Using the way of “word cloud” is depicting the frequency of drugs PGx detected from the institutions of participants (Pandya and Bajorek, 2017).

## Results

### General Profiles of Responders

**FIGURE 1:**
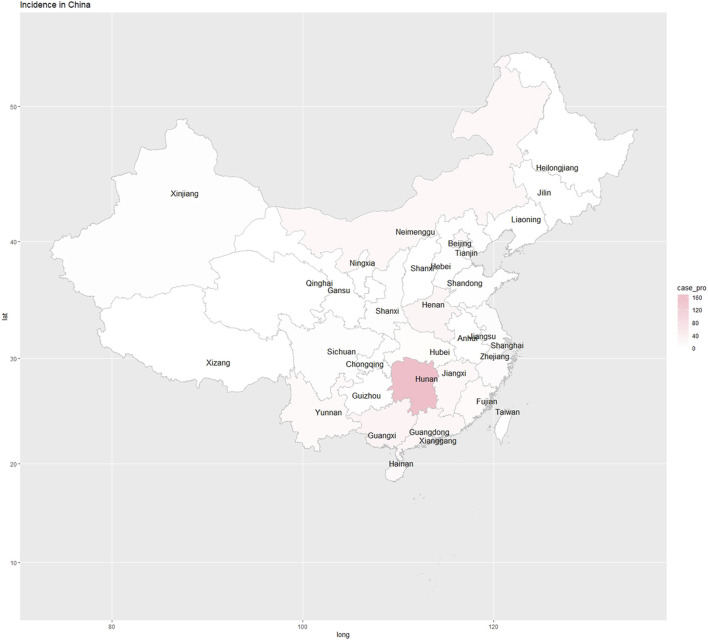
An overall map of PGx participants were from all provinces, municipalities, and autonomous regions.

Of the 918 participants in the survey, 422 were eligible for inclusion based on their career information. Four hundred and twenty-two participants from 25 provinces, municipalities, and autonomous regions responded to this survey (Figure 1). As shown in Table 1, these participants were categorized as physicians (n = 117, 27.7%), pharmacists (n = 132, 31.3%), and researchers (n = 173, 41.0%). There were 202 males (47.9%) and 220 females (52.1%). Most participants were aged between 20 and 49 years old with an education level of college and above.

**TABLE 1:** Characteristics of responders.

Characteristic	Physicians	Pharmacists	Researchers	Combined
N	117 (27.7%)	132 (31.3%)	173 (41.0%)	422
Gender				
Male	70 (59.8%)	44 (33.3%)	88 (50.9%)	202(47.9%)
Female	47 (40.2%)	88 (66.7%)	85 (49.1%)	220(52.1%)
Age group				422
<20	1 (0.9%)	0 (0%)	3 (1.7%)	4(1.0%)
20–29	21 (17.9%)	38 (28.8%)	87 (50.3%)	146(34.6%)
30–39	66 (56.4%)	73 (55.3%)	64 (37.0%)	203(48.1%)
40–49	23 (19.7%)	18 (13.6%)	12 (6.9%)	53(12.6%)
50–59	6 (5.1%)	3 (2.3%)	7 (4.0%)	16(3.8%)
≥60	0 (0%)	0(0%)	0(0%)	0(0%)
Educational level				
Below bachelor	6 (5.1%)	0	2 (1.2%)	8(1.9%)
Bachelor	66 (56.4%)	29 (22.0%)	36 (20.8%)	131(31.0%)
Master	31 (26.5%)	71 (53.8%)	82 (47.4%)	184(43.6%)
PhD	14 (12.0%)	32 (24.2%)	53 (30.6%)	99(23.5%)

### Awareness of Pharmacogenomics

**FIGURE 2:**
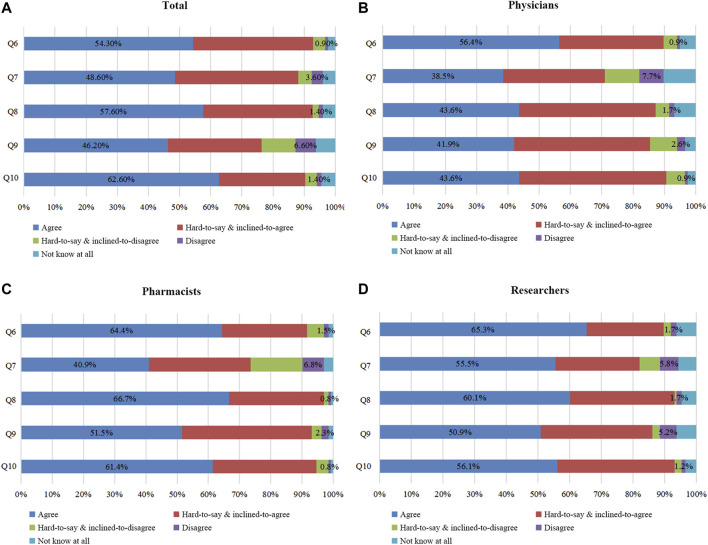
Awareness survey of PGx among all respondents. Figure 2A: All participants’ awareness surveys on questions (Q) 6–10. Figures 2B–D: Q6–10 were assessed by physicians, pharmacists, and researchers. Q6 In your opinion, is PGx capable of aiding in patient selections of optimal drugs? Q7 In your opinion, can PGx assist patients in using correct doses? Q8 In your opinion, can PGx assist patients in preventing severe adverse reactions? Q9 In your opinion, can PGx DNA detection lower the economic burdens and save medical costs for patients? Q10 In your opinion, should clinical application of PGx DNA detection be further promoted?

To survey the status of PGx awareness in different institutes and hospitals, we firstly asked the following questions: Q6, whether PGx assists in selecting optimal drugs for patients; Q7, whether PGx assists patients in using correct doses; and Q8, whether PGx assists patients in preventing the occurrences of severe adverse reactions. The awareness (agree) level of PGx among all responders was 54.3, 48.6, and 57.6%, respectively (Figure 2A). With the highest awareness of PGx capable of predicting adverse drug reactions, the responders tended to have the lowest awareness of PGx for guiding drug selections. Among the three groups (Figures 2B–D), pharmacists had the highest awareness of PGx’s impact. Their answers to all three questions were positive (>50.0%) at 61.4, 51.5, and 66.7% respectively. As for researchers, the awareness levels were lower; however, all of their answers were also positive at 56.1, 50.9, and 60.1% respectively. The awareness levels were the lowest among the physicians whose answers were overall negative (<50.0%) at 43.6, 41.9, and 43.6%, respectively. Table 2, Table 3 show the results of comparing the three groups using chi-square test and logistic regression analysis. For Q6, statistical differences using chi-square test existed in approval rates among the three groups (*p* = 0.016); the multivariate analysis using logistic regression method for the awareness levels in Q6 among the three groups indicated that pharmacists (OR: 1.783; 95% CI: 1.033–3.076; *p* = 0.038) and researchers (OR: 1.589; 95% CI: 0.935–2.702; *p* = 0.087). For Q7, no statistical differences existed among the three groups (*p* = 0.232); the multivariate analysis for the awareness levels in Q7 showed that pharmacists (OR: 1.242; 95% CI: 0.718–2.149; *p* = 0.438) and researchers (OR: 1.426; 95% CI: 0.833–2.439; *p* = 0.195). There were also statistical differences in agreement rates among the three groups when they answered Q8 (*p* = 0.001); the multivariate analysis for the awareness levels in Q8 revealed that pharmacists (OR: 2.199; 95% CI: 1.262–3.834; *p* = 0.005) and researchers (OR: 1.872; 95% CI: 1.093–3.208; *p* = 0.022).

**TABLE 2:** Awareness of PGx (*χ* ^2^ -test).

Survey question	AgreeN (%)	Not agreeN (%)	Combined*P*
Q6: In your opinion, is PGx capable of aiding in patient selections of optimal drugs?			
Physicians	51 (43.6%)	66 (56.4%)	0.016
Pharmacists	81 (61.4%)	51 (38.6%)	
Researchers	97 (56.1%)	76 (43.9%)	
Q7: In your opinion, can PGx assist patients in using correct doses?			
Physicians	49 (41.9%)	68 (58.1%)	0.232
Pharmacists	68 (51.5%)	64 (48.5%)	
Researchers	88 (50.9%)	85 (49.1%)	
Q8: In your opinion, can PGx assist patients in preventing severe adverse reactions?			
Physicians	51 (43.6%)	66 (56.4%)	0.001
Pharmacists	88 (66.7%)	44 (33.3%)	
Researchers	104 (60.1%)	69 (39.9%)	
Q9:In your opinion, can PGx DNA detection lower the economic burdens and save medical costs for patients?			
Physicians	45 (38.5%)	72 (61.5%)	0.006
Pharmacists	54 (40.9%)	78 (59.1%)	
Researchers	96 (55.5%)	77 (44.5%)	

**TABLE 3:** Awareness of PGx (multivariate logistic regression).

Survey response	Adjusted OR (95% CI)	*p* value
Q6: In your opinion, is PGx capable of aiding in patient selections of optimal drugs?		
Occupation		
Pharmacists	1.783 (1.033–3.076)	0.038
Researchers	1.589 (0.935–2.702)	0.087
Physicians	1.0	
Educational level		
PhD	1.339 (0.300–5.986)	0.702
Master	1.350 (0.309–5.901)	0.690
Bachelor	0.693 (0.161–2.989)	0.623
Below bachelor	1.0	
Age group	1.471 (1.119–1.933)	0.006
Q7: In your opinion, can PGx assist patients in using correct doses?		
Occupation		
Pharmacists	1.242 (0.718–2.149)	0.438
Researchers	1.426 (0.833–2.439)	0.195
Physicians	1.0	
Educational level		
PhD	1.042 (0.229–4.733)	0.958
Master	1.501 (0.337–6.689)	0.594
Bachelor	0.594 (0.135–2.614)	0.491
Below bachelor	1.0	
Age group	1.663 (1.262–2.192)	<0.001
Q8: In your opinion, can PGx assist patients in preventing severe adverse reactions?		
Occupation		
Pharmacists	2.199 (1.262–3.834)	0.005
Researchers	1.872 (1.093–3.208)	0.022
Physicians	1.0	
Educational level		
PhD	1.541 (0.340–6.982)	0.575
Master	1.531 (0.346–6.771)	0.574
Bachelor	0.692 (0.159–3.017)	0.624
Below bachelor	1.0	
Age group	1.573 (1.186–2.088)	0.002
Q9: In your opinion, can PGx DNA detection lower the economic burdens and save medical costs for patients?		
Occupation		
Pharmacists	1.114 (0.643–1.930)	0.701
Researchers	2.298 (1.343–3.933)	0.002
Physicians	1.0	
Educational level		
PhD	0.975 (0.216–4.405)	0.974
Master	0.931 (0.210–4.119)	0.925
Bachelor	0.723 (0.166–3.155)	0.666
Below bachelor	1.0	
Age group	1.546 (1.176–2.032)	0.002

Only 46.2% of responders felt that PGx DNA detection lowered the economic burdens of patients (Q9) (Figure 2A). As shown in Figures 2B–D, 38.5% of physicians and 40.9% of pharmacists had this answer. The overall attitude for both physicians and pharmacists was thus negative. Only researchers were positive (55.5%). As Table 2, Table 3 the agreement rates among the three groups were statistically different (*p* = 0.006); further multivariate logistic regression analysis revealed that pharmacists (OR: 1.114; 95% CI: 0.643–1.930; *p* = 0.701) and researchers (OR: 2.298; 95% CI: 1.343–3.933; *p* = 0.0.002).

Regarding Q10 (“whether the clinical application of PGx DNA detection should be promoted”), the agreement level was as high as 62.6% (Figure 2A). The agreement level was 56.4, 64.4, and 65.3% among physicians, pharmacists, and researchers respectively (Figures 2B–D).

### Hindering Factors of Pharmacogenomics Implementation

**FIGURE 3:**
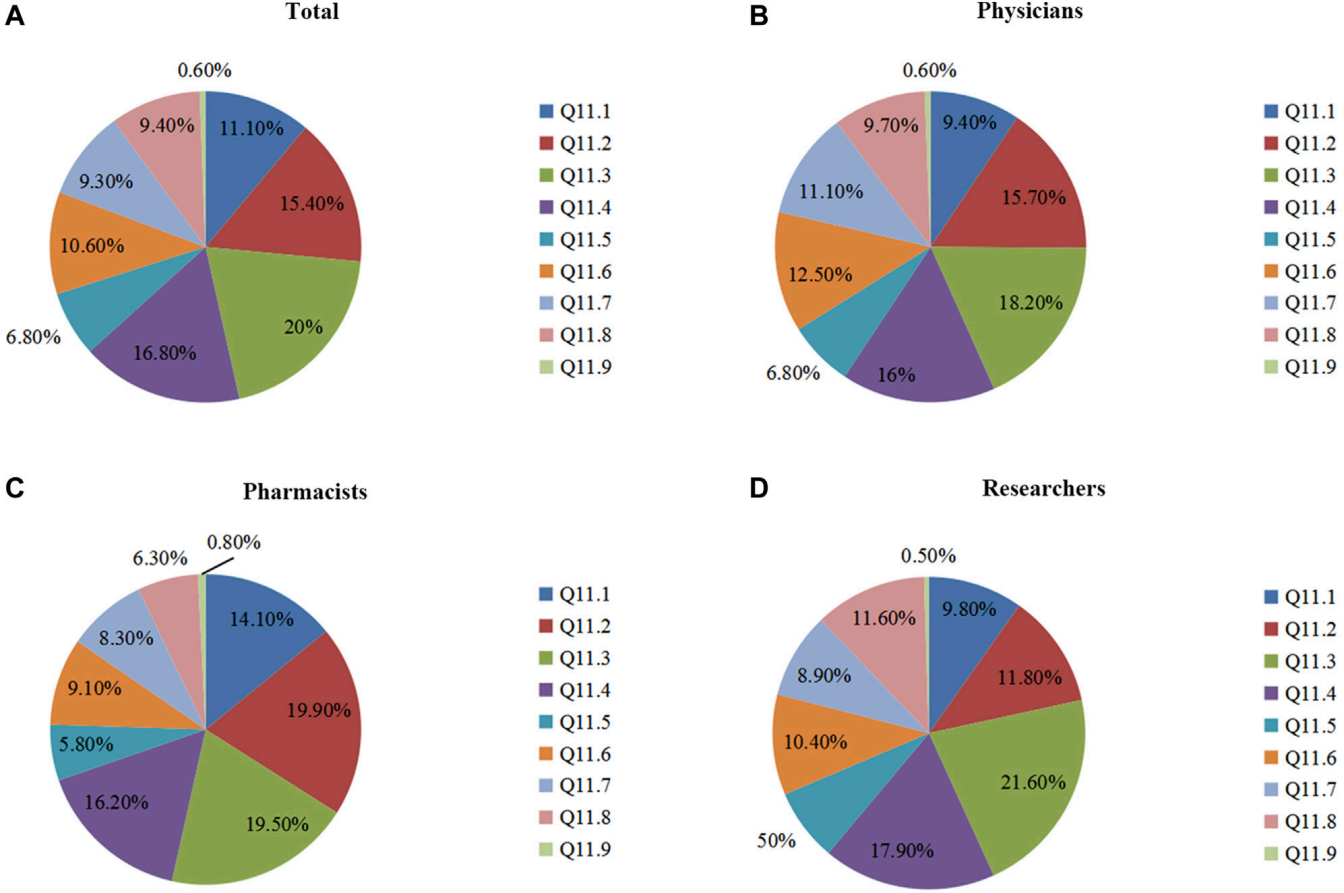
The survey of major influencing factors of PGx’s clinical application. Figure 3A: Assessment of all participants’ understanding of Q 11. Figures 3B–D: Understanding of Q 11 by physicians, pharmacists, and researchers. Q11 In your opinion, which were three major influencing factors of PGx’s clinical application (selecting three options)? 11.1 Not including into clinical detecting catalogue of National Health and Population Control Commission. 11.2 Lacking an application guideline of PGx specifically for Chinese patients. 11.3 Lacking sector codes of PGx’s clinical application. 11.4 Lacking large-scaled clinical trials of PGx. 11.5 Lacking a pricing standard for PGx DNA detection. 11.6 Lacking a reporting standardization for PGx DNA detection. 11.7 Not including PGx DNA detection into National Medical Insurance Scheme. 11.8 Not knowing PGx. 11.9 Miscellaneous?

In Q11, we surveyed the current major factors hindering PGx’s clinical implementation. Among all of the responders, the top three factors were lacking sector standards for PGx's clinical application (20.0%), lacking large-scale clinical trials of PGx (16.8%), and lacking a Chinese application guideline for PGx (15.4%) (Figure 3A). Although there were small differences, these three factors were also considered as having major impacts upon the clinical applications of PGx across the three groups (Figures 3B–D). Other influencing factors of PGx’s clinical application included: PGx tests not included in the National Health Commission's catalog of clinical detection (11.1%), lacking reporting standardization for PGx DNA detection (10.6%), not knowing PGx at all (9.4%), not including PGx DNA detection in the National Medical Insurance Scheme (9.3%), and lacking a pricing criterion for PGx DNA detection (6.8%).

### Promoting the Implementation of Pharmacogenomics

**FIGURE 4:**
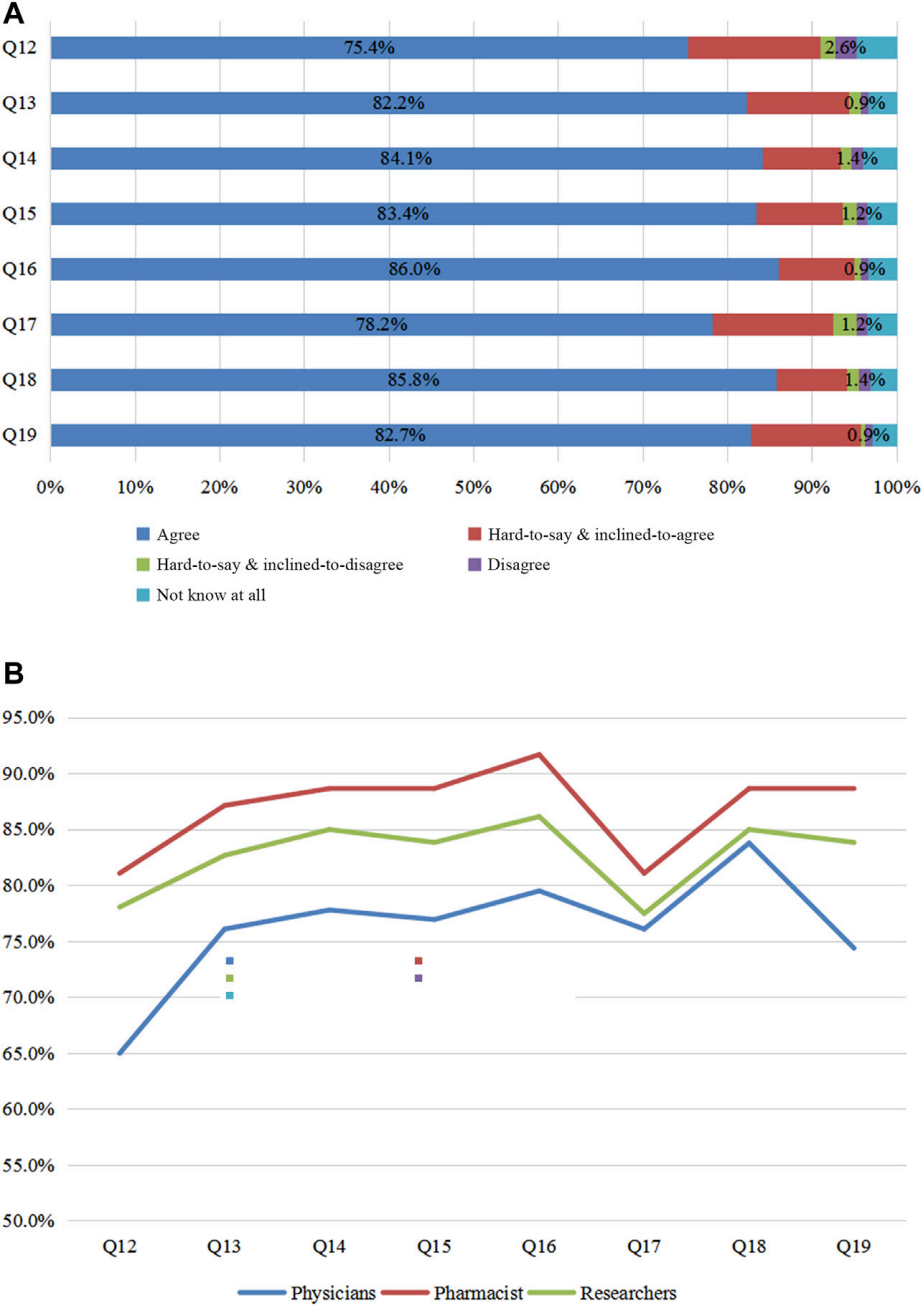
Survey of promoting the application of PGx. Figure 4A: Views of all participants on Q 12–19. Figure 4B: Questions 12–19 were considered by physicians, pharmacists, and researchers. Q12 In your opinion, should the government formulate the relevant regulations for PGx DNA detection? Q13 In your opinion, is it necessary to formulate the relevant guideline of individualized PGx therapy for Chinese populations? Q14 In your opinion, is it necessary to formulate the sector codes of PGx DNA detection? Q15 In your opinion, is it necessary to formulate the pricing criteria for PGx DNA detection? Q16 In your opinion, is it necessary to formulate the reporting standardization of PGx DNA detection? Q17 In your opinion, should PGx DNA detection be included into National Medical Insurance Scheme? Q18 In your opinion, is it necessary to establish the PGx knowledge database for Chinese populations? Q19 In your opinion, should PGx courses be offered at colleges?

The following eight possible drivers of PGx’s clinical application were surveyed: Q12 (“whether formulating the relevant regulations of PGx DNA detection,”) Q13 (“whether formulating the guidelines for individualized PGx-based therapies for Chinese populations”), Q14 (“whether formulating the sector standards of PGx DNA detection”; sector standards are standards formulated in accordance with uniform technical requirements within a certain discipline), Q15 (“whether formulating the pricing criteria of PGx DNA detection”), Q16 (“whether formulating the reporting standardization of PGx DNA detection”), Q17 (“whether including PGx DNA detection in the National Medical Insurance Scheme”), Q18 (“whether establishing a PGx-based knowledge database for Chinese populations”), and Q19 (“whether offering the PGx–related courses at colleges”). Over 70% of responders were positive for the approving options (Figure 4A). In their opinions, it was imperative to formulate the relevant regulations, guidelines, sector standards, pricing criteria, reporting standardization, knowledge database, and PGx detection in the National Medical Insurance Scheme and to offer related courses at colleges. Among the three groups (Figure 4B), pharmacists agreed on the above eight measures (81.8, 87.9, 88.6, 88.6, 91.7, 81.1, 88.6, 88.6%) more than researchers (78, 82.7, 85, 83.8, 86.1, 77.5, 85, 83.8%) and physicians (65, 76.1, 77.8, 76.9, 79.5, 76.1, 83.8, 74.4%) did. The data suggest that pharmacists were the most eager to anticipate further improvements for PGx’s clinical application.

### Current Status of Implementing Pharmacogenomics Testing

**FIGURE 5:**
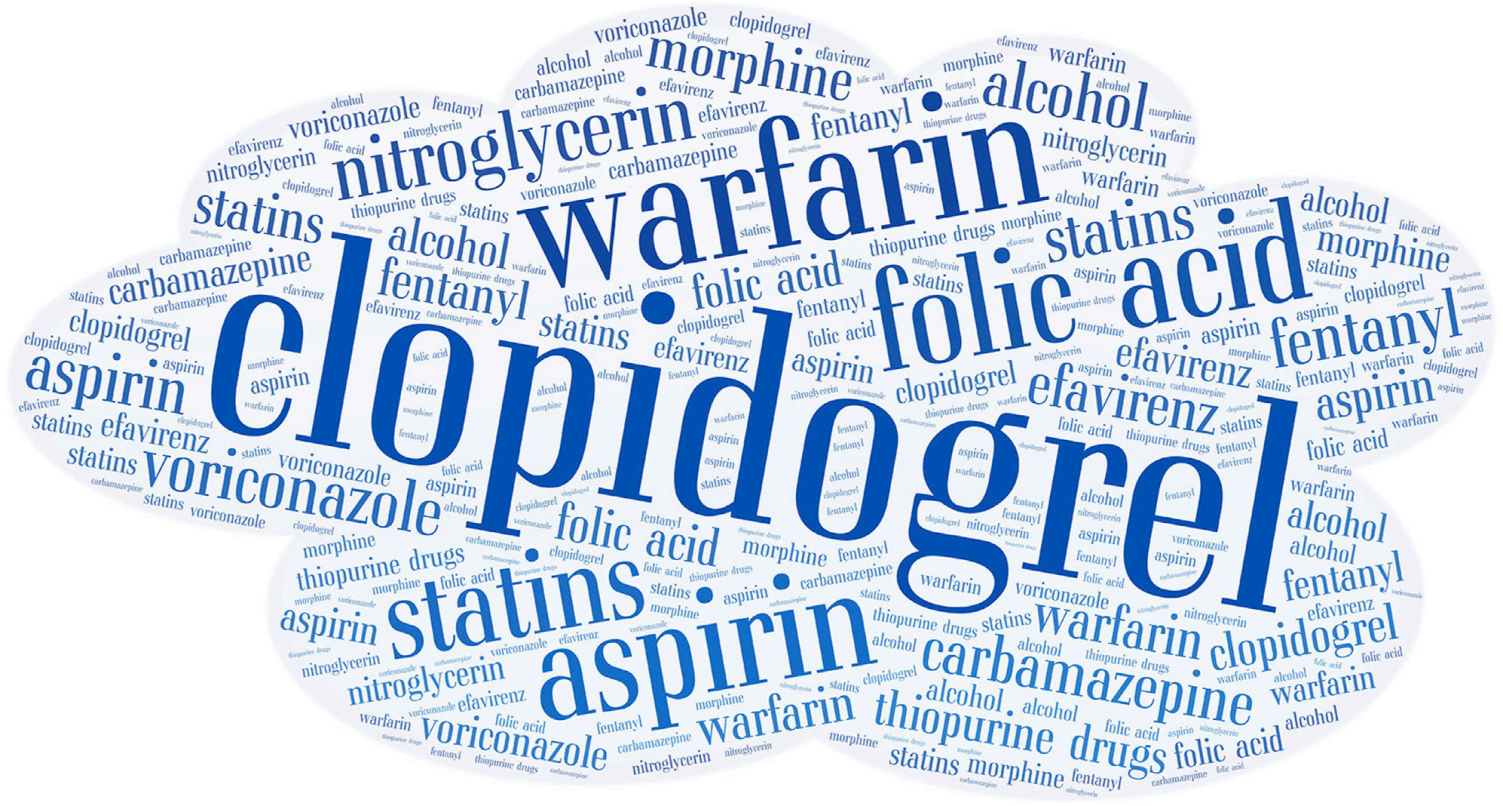
Word cloud depicted the frequency of drugs detected by PGx from the institutions of participants. The size of the print is proportional to the frequency of drug detection.

The institutes of participants were also surveyed for the presence or absence of PGx testing (Q20). As shown in Figure 5, a PGx test was conducted for a variety of drugs including warfarin, clopidogrel, statins, nitroglycerin, folic acid, carbamazepine, aspirin, morphine, fentanyl, efavirenz, voriconazole, and thiopurine. The most common drugs tested were warfarin and clopidogrel.

## Discussion

The clinical application of PGx has been gradually known in mainland China. To assess the status of PGx’s clinical application, we conducted this questionnaire survey among physicians, pharmacists, and researchers in mainland China. While we found that the agreement level of PGx implementation was less than 50% in physicians, over 50% of both pharmacists and researchers showed a positive attitude toward PGx testing.

A United States survey indicated that 97.6% of physicians believed that hereditary factors could influence drug therapies, while a Dutch survey of pharmacists revealed that 99.7% of responders thought that drug therapies could be affected by patient gene profiles (Stanek et al., 2012; Bank et al., 2017). Our survey results indicated that Chinese physicians and pharmacists had a lower awareness level of hereditary factors influencing drug responses. Furthermore, most physicians and pharmacists surveyed did not agree that PGx DNA detection could lower the economic burdens for patients. In China, there is currently no specific pricing criterion for PGx DNA detection. Many institutes normally charge a rate of RMB 300 per mutation locus, and the cost of PGx testing for a drug is close to RMB 1,000. For example, two loci (CYP2C9*3, VKORC1 -1639G>A) of genetic tests are usually required for warfarin therapy. Thus the minimal charge is RMB 600. As for genetic testing of another common drug clopidogrel, three loci of CYP2C19*2, CYP2C19*3, and CYP2C19*17 are usually required with a minimal charge of RMB 900. If genetic tests were needed for multiple drugs, the total charge could easily go up to thousands or even tens of thousands of Yuan. The genetic test cost of drug-gene pairs may greatly surpass the fees of purchasing the drugs or using alternative agents. As a result, both physicians and pharmacists may have a bias toward PGx due to a high price tag and a potential low ratio of cost-effectiveness. Although physicians (56.4%), pharmacists (64.4%), and researchers (65.3%) advocated the clinical implementation of PGx tests, the positive attitude was less than 70% among them. The public awareness level of PGx remains low in China. Conceptually, many people feel that PGx might add medical costs. There is a lack of pharmacoeconomic analysis on PGx in China. Such data may be critical to the education of healthcare providers in promotion of PGx application in China.

Three major factors influencing PGx’s clinical application in China were further identified: “lacking a sector standard of PGx’s clinical application,” “lacking large-scaled PGx-based clinical trials,” and “lacking a national guideline of PGx’s application for Chinese patients." To our knowledge, the existing pharmacogenomics questionnaires do not address in detail the factors affecting the clinical application of pharmacogenomics. However, this is a relatively important topic. Some researchers believe that the testing of pharmacogenomics, including testing technology, test result report and testing cost, is the main challenge of the clinical application of pharmacogenomics (Moyer and Caraballo, 2017). However, with the development and application of sequencing technology, the detection of PGx is becoming easier. The test report has been covered in our questionnaire and is also considered an important factor affecting the application of pharmacogenomics.

Overall, the majority of responders were optimistic on the future of PGx in China. However, they agreed that, to effectively implement PGx in China, it was imperative to establish the PGx-related regulations, guidelines, sector standards, reporting standardization, and knowledge database. Moreover, PGx testing should be included in the National Medical Insurance Scheme and the related tutoring courses offered at colleges. The stated mission of the Division of Pharmacogenomics of the Chinese Pharmacological Society has been advancing the research and development of PGx in the mainland of China. This national organization is also striving to accelerate the research of PGx and to formulate relevant application guidelines. In addition, many PGx-themed groups in local or provincial scientific organizations have been formed to strengthen the research and applications of PGx. For example, Hunan Provincial Medical Association has a subcommittee of Translational Pharmacogenomics and the Pharmaceutical Associations at the provinces of Hunan, Jiangsu, and Anhui have a special committee of PGx (Jiangsu Pharmaceutical Association, 2015; Anhui Pharmaceutical Association, 2019). A PGx database has been established (http://www.chnpgxc.com/). The mission of this online resource is to establish a knowledge center of PGx, formulate the sector standard and application guidelines for PGx, construct an overall system of decision-making supports and education, offer recommendation on individualized disease therapies, and provide precise drug discovery and development. It is believed that an increasing number of PGx researchers and other stakeholders will propel the future development of PGx in the mainland of China.

PGx tests have been recommended and provided by some institutes for a variety of drugs that include cardiovascular agents (warfarin, clopidogrel, statins, nitroglycerin, folic acid and aspirin), antiepileptic agents (carbamazepine), analgesics and anesthetics (morphine and fentanyl), antibiotics (efavirenz, voriconazole), and antineoplastics agents (thiopurines). In our survey, some responders did not give specific drugs for which a PGx test was recommended in their institutes; instead, they only listed drug categories such as antineoplastics, anti-platelet, hypotensive, and antipsychotic agents. The information regarding PGx testing on specific drugs was rather limited in the present study. Furthermore, our survey targeted three groups of healthcare providers including physicians, pharmacists, and researchers. The employees of third-party gene detection agencies and other stakeholders were not included. At present, most PGx tests are provided by third-party testing agencies, while hospital participation is relatively limited. This is particularly true for the antineoplastics of targeted therapy where PGx tests are often critical to a satisfying treatment. As a result, it might be one of the reasons why antineoplastics agents were not a top list by the responders in our survey.

Our survey has some restrictions. Firstly, a new survey mode is adopted through an online platform of Wenjuanxing and WeChat. It is simple and easy to implement and data collecting and survey completing may be finished in the shortest time. However, it also has some shortcomings. For example, it is not known how many people have accessed the survey link. Only those interested professionals fill out the questionnaire. This leads to an inability of obtaining response rates. Secondly, our research is mainly to explore the understanding of physicians, pharmacists and researchers regarding pharmacogenomics. All these responders are highly educated. In addition, pharmacogenomics course is not taught among undergraduates. Only some universities take pharmacogenomics as an elective course in postgraduate studies. It may explain high educational background in our survey. Therefore this result is representative of clinicians and pharmacists practicing PGx in china. In the future, we will expand the investigation of pharmacogenomics to general population so as to obtain a more comprehensive understanding of pharmacogenomics in Chinese population. Thirdly, implementation and factors affecting implementation may vary across diverse practicing settings so it would be useful to include a question Q20 on the respondent’s practice setting. Unfortunately few people have answered it probably because of privacy or some other unknown reason. Fourthly, although we attempted to assess the influencing factors for applying pharmacogenomics as comprehensively as possible, more insightful information is desirable. For example, it is whether the interviewees have prior training or education related to pharmacogenomics. Even if we recognize this deficiency, we regret failing to trace back to the pharmacogenomics education/training background of each respondent population due to the anonymous nature of our survey.

## Conclusion

A survey of PGx knowledge and application was conducted among physicians, pharmacists, and researchers in China. We found that a significant number of healthcare providers, in particular physicians, remain unable to recognize the importance of PGx in drug therapy. Our results have offered information on the current status of PGx application in China. Although PGx research has been advanced rapidly in recent years in the mainland of China, the clinical implementation of PGx has a long way to go. Guo et al., 2019.

## Data Availability

The original contribution presented in the study are included in the article/Supplementary Material, further inquiries can be directed to the corresponding authors.
