# Supplementary material for: A Survey of Pharmacogenomics Testing Among Physicians, Pharmacists, and Researchers From China
Source: Front Pharmacol. 2021 Jul 12;12:682020. doi: 10.3389/fphar.2021.682020 (PMC8311355; doi:10.3389/fphar.2021.682020)
Supplement: Supplementary file 1 [file Table1.DOCX]

**Questionnaire of PGx’s Knowledge Proficiency**

Initiated by Clinical Pharmacology Center, Third Xiangya Hospital, Central South University, this anonymous questionnaire is designed for evaluating the current knowledge proficiency of pharmacogenomics (PGx). However, the results of this survey shall be publicized in scientific journals for the references of PGx researchers. We greatly appreciate your precious time in responding to the following questions.

Q1. Your gender:

Male

Female

Q2. Your age group:

<20 yr

20-29 yr

30-39 yr

40-49 yr

50-59 yr

≥60 yr

Q3. Your educational level:

Below bachelor

Bachelor

Master

PhD

Q4. Your occupation:

Physician

Pharmacist

Researcher

Miscellaneous

Q5. Your residency locality:

Q6. In your opinion, is PGx capable of aiding in patient selections of optimal drugs?

0: Uncertain;

1: Disagree;

2: Undecided but inclined to disagree;

3: Undecided but inclined to agree;

4: Agree

Q7. In your opinion, can PGx assist patients in using correct doses?

Q8. In your opinion, can PGx assist patients in preventing severe adverse reactions?

Q9. In your opinion, can PGx DNA detection lower the economic burdens and save medical costs for patients?

Q10. In your opinion, should clinical application of PGx DNA detection be further promoted?

Q11. In your opinion, which were three major influencing factors of PGx’s clinical application (selecting three options)?

11.1 Not including into clinical detecting catalogue of National Health & Population Control Commission

11.2 Lacking an application guideline of PGx specifically for Chinese patients

11.3 Lacking sector codes of PGx’s clinical application

11.4 Lacking large-scaled clinical trials of PGx

11.5 Lacking a pricing standard for PGx DNA detection

11.6 Lacking a reporting standardization for PGx DNA detection

11.7 Not including PGx DNA detection into National Medical Insurance Scheme

11.8 Not knowing PGx

11.9 Miscellaneous

Q12. In your opinion, should the government formulate the relevant regulations for PGx DNA detection?

Q13. In your opinion, is it necessary to formulate the relevant guideline of individualized PGx therapy for Chinese populations?

Q14. In your opinion, is it necessary to formulate the sector codes of PGx DNA detection?

Q15. In your opinion, is it necessary to formulate the pricing criteria for PGx DNA detection?

Q16. In your opinion, is it necessary to formulate the reporting standardization of PGx DNA detection?

Q17. In your opinion, should PGx DNA detection be included into National Medical Insurance Scheme?

Q18. In your opinion, is it necessary to establish the PGx knowledge database for Chinese populations?

Q19. In your opinion, should PGx courses be offered at colleges?

Q20. At your institution, is PGx DNA detecting implemented? If yes, which gene(s) and drug(s) are detected? What is your professional affiliation? Are there other recommendations for implementing PGx detection?

Your precious time of participating in this survey is great appreciated again. If there is any doubt or suggestion regarding this questionnaire or study, please send email to us (1938885164@qq.com). And staff of PGx Database (http://www.chnpgxc.com) offered generous guidance for this project.
